# Royal Westminster Ophthalmic Hospital

**Published:** 1902-03-15

**Authors:** 


### ROYAL WESTMINSTER OPHTHALMIC HOSPITAL.

Though the secretary was heard to congratulate himself on the large attendance at the meeting on Friday last week, it was evident that tea had a greater attraction for the governors and subscribers than an annual general meeting. To the reception, at 4.30, came quite a crowd, while the meeting, half an hour earlier, was attended by only two or three subscribers over and above the committee. There were 15 altogether. The chairman, Sir Charles A. Turner, K.C.I.E., presided, and the report was unanimously adopted.

The main point of interest in the report for 1901 is the reference to the new ophthalmic department at Charing Cross Hospital. This department, the report says, has relieved the Royal Westminster Ophthalmic Hospital from the obligation of teaching the students of the Charing Cross Medical School, and this will enable it to adapt the curriculum more closely to the needs of qualified medical men. This, and the increased facilities afforded by the recent remodelling of the hospital, will, it is thought, eventually largely increase the number of students. In view of the new arrangements announced for Army medical officers, it is believed that the usefulness of the School will increase.

The question of rebuilding, which has been under consideration for some years, has been temporarily shelved from lack of means. The appeal issued by the committee came before the public at the same moment as other appeals from larger hospitals, and it appeared certain that many years would elapse before a sufficient sum could be accumulated to justify the acquisition of a new site and the erection of a new hospital. The larger project has therefore been postponed, and the present building has been remodelled and improved at a cost of about £9,000. Mr. W. Adams Frost, F.R.C.S., one of the surgeons of the hospital, has given the whole of the appliances in the operating-theatre. Sums from the amount contributed for building (£3,305) have only been drawn upon with the consent of the donors, but about £2,635, which had been contributed by the General Purposes Fund to the Building Fund has been withdrawn, and securities representing this amount will be sold at a favourable opportunity. To meet the balance of the expenditure incurred, the committee earnestly appeal to the generosity of the public.

